# Text Messaging to Improve Linkage, Retention, and Health Outcomes Among HIV-Positive Young Transgender Women: Protocol for a Randomized Controlled Trial (Text Me, Girl!)

**DOI:** 10.2196/12837

**Published:** 2019-07-29

**Authors:** Cathy J Reback, Jesse B Fletcher, Anne E Fehrenbacher, Kimberly Kisler

**Affiliations:** 1 Friends Research Institute, Inc Los Angeles, CA United States; 2 Department of Psychiatry and Biobehavioral Sciences University of California, Los Angeles Los Angeles, CA United States

**Keywords:** HIV, AIDS, transgender persons, text messaging

## Abstract

**Background:**

Transgender women in the United States experience numerous risk factors for HIV acquisition and transmission, including increased rates of homelessness, alcohol and drug use, sex work, and nonprescribed hormone and soft tissue–filler injections. In addition, transgender women face discrimination and social/economic marginalization more intense and deleterious than that experienced by lesbian, gay, or bisexual individuals, further worsening health outcomes. Although little research has been done specifically with young transgender women aged 35 years and younger, existing evidence suggests even further elevated rates of homelessness, substance use, and engagement in HIV transmission risk behaviors relative to their older transgender women and nontransgender young adult counterparts. Young transgender women living with HIV experience a range of barriers that challenge their ability to be successfully linked and retained in HIV care.

**Objective:**

The aim of this randomized controlled trial, *Text Me, Girl!*, is to assess the impact of a 90-day, theory-based, transgender-specific, text-messaging intervention designed to improve HIV-related health outcomes along the HIV care continuum among young (aged 18-34 years) transgender women (N=130) living with HIV/AIDS.

**Methods:**

Participants were randomized into either Group A (immediate text message intervention delivery; n=61) or Group B (delayed text message intervention delivery whereby participants were delivered the text-messaging intervention after a 90-day delay period; n=69). Over the course of the 90-day intervention, participants received 270 theory-based text messages that were targeted, tailored, and personalized specifically for young transgender women living with HIV. Participants received 3 messages per day in real time within a 10-hour gradual and automated delivery system. The text-message content was scripted along the HIV care continuum and based on social support theory, social cognitive theory, and health belief model. The desired outcome of *Text Me, Girl!* was virological suppression.

**Results:**

Recruitment began on November 18, 2016, and the first participant was enrolled on December 16, 2016; enrollment closed on May 31, 2018. Intervention delivery ended on November 30, 2018, and follow-up evaluations will conclude on August 31, 2019. Primary outcome analyses will begin immediately following the conclusion of the follow-up evaluations.

**Conclusions:**

Text messaging is a communication platform well suited for engaging young transgender women in HIV care because it is easily accessible and widely used, as well as private, portable, and inexpensive. *Text Me, Girl!* aimed to improve HIV care continuum outcomes among young transgender women by providing culturally responsive text messages to promote linkage, retention, and adherence, with the ultimate goal of achieving viral suppression. The *Text Me, Girl!* text message library is readily scalable and can be adapted for other hard-to-reach populations.

**International Registered Report Identifier (IRRID):**

DERR1-10.2196/12837

## Introduction

As a population, transgender women bear a disproportionate burden of HIV/AIDS and other health disparities. In the United States, transgender women experience numerous risk factors for HIV acquisition and transmission [1-3], including increased rates of homelessness [4-6], alcohol and drug use [6,7], and sex work [7,8]. In addition, many transgender women use nonprescribed hormone injections and/or medically unsupervised soft tissue–filler injections, HIV risk factors that are specific to this population [5,9,10]. Furthermore, transgender women face discrimination and social/economic marginalization more intense and deleterious than that experienced by lesbian, gay, or bisexual individuals [4], further worsening expected health outcomes [11-13].

Although HIV surveillance data are often not collected for transgender persons in the United States [14], meta-analytic and aggregated jurisdictional data suggest that HIV prevalence rates among transgender women are an order of magnitude higher than the US general population (18.4%-30.6% [7,15] vs 0.3%-0.4% [16]), with the odds of becoming HIV-positive estimated to be 34.2 times higher for transgender women than other US adult populations [15]. Within Los Angeles county (LAC), there is a stark disparity such that transgender women exhibit the second highest estimated HIV prevalence rate of any priority group (16.7%), marginally lower than the prevalence for men who have sex with men (MSM) (18.4%) [17]. A community-based convenience sample of transgender women in LAC evidenced an increase in self-reported HIV prevalence of close to 60% over a 17-year period, from 22% in 1998-1999 to 35% in 2015-2016 [18]. Although transgender women make up only 0.1% of the general population in LAC, they represent 1.3% of all people living with HIV (PLWH) and 1.4% of recent diagnoses from 2009 to 2013 [19]. A study found that more than half of the respondents reported recent homelessness, less than a third were employed, more than two-thirds reported doing sex work in the past 3 months, 85% reported HIV transmission risk behaviors, and 20% were HIV-positive [20]. As a result, transgender women have a nearly 7 times higher likelihood in delaying medical care after an HIV-positive diagnosis than cisgender women [21]. Multilayered stigma, accompanied by an inability to pay for care and misinformation about both the need for antiretroviral therapy (ART) and/or the existence of ART/hormone therapy interactions, often contributes to low linkage to and retention in care outcomes among young transgender women [22,23].

With the high HIV prevalence rate among transgender women, it is necessary to develop interventions to improve linkage to ART and address the multitude of barriers experienced by transgender women to enhance their retention in HIV care. A potential solution to improve adherence and retention among transgender women is through mobile phone text messages. Several studies have found success in improving health services and outcomes through the use of text message interventions with PLWH [24-28]. In 2014, internet and mobile phone use among people in the United States aged 12 to 29 years was over 90% [29]. Unlike internet-based social media platforms, text messaging is easily accessible, private, portable, inexpensive, and used daily by nearly all the youth and the young adults in the United States, including the most vulnerable transgender women [30-32]. Text messaging is an appropriate modality for reaching young transgender women because this population regularly uses text messaging and social media platforms to gain positive perspectives on their gender identity, develop Web-based communities, gather health information, purchase gender-confirmation hormones, and acquire sex partners [33-36].

In regard to HIV care, a study with PLWH found that a personalized daily text message reminder improved participants’ adherence to ART compared with those who received a beeper reminder [24]. Likewise, in another trial, youth living with HIV who had poor adherence reported better self-management of their HIV medication after 3 months when receiving culturally relevant and motivational daily text messages [27]. Given the rapid increase of mobile phone use and its accessibility to transgender women, it is important to understand the significance of text message use in facilitating HIV care among this high-risk population. Therefore, the purpose of this study was to determine whether providing culturally responsive text messages that promote healthy living and linkage, retention, and adherence may help young transgender women living with HIV in LAC be successfully linked and retained in HIV care with the ultimate goal of viral suppression.

## Methods

### Study Design

**Figure 1:**
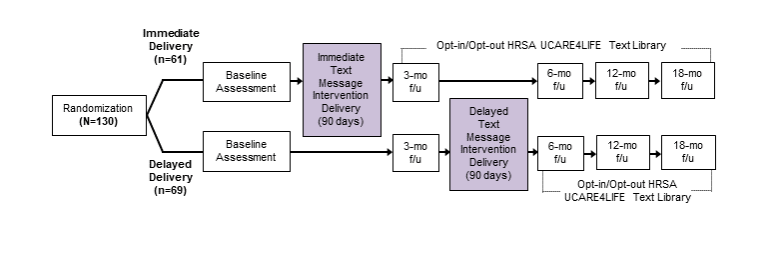
*Text Me, Girl!* study design. f/u: follow-up; HRSA: Health Resources and Services Administration; mo: month.

*Text Me, Girl!* was a randomized controlled trial to assess the impact of a 90-day theory-based, transspecific, text-messaging intervention designed to improve health outcomes along the HIV care continuum, with the desired outcome of viral suppression among HIV-positive young transgender women (Figure 1). The HIV care continuum is a model used to identify gaps in HIV services and develop strategies to improve engagement in care among PLWH. The HIV care continuum outlines sequential stages as follows: diagnosis of HIV infection, linkage to care, retention to care, adherence to ART therapy, and achievement of viral suppression [37]. All study procedures were approved by the Friends Research Institute (FRI) Institutional Review Board and the Western Institutional Review Board.

During the 90-day intervention period, participants received 3 scripted, theory-based, transspecific text messages per day (270 text messages total), which were targeted, tailored, and personalized. The *Text Me, Girl!* text message library was adapted from a larger library of 616 messages originally developed for a study with methamphetamine-using MSM [38].

Only the messages related to the HIV care continuum were modified: (1) HIV positivity/physical and emotional health, (2) linkage/retention in HIV care, and (3) ART medication adherence/viral load suppression. The text message library was tailored specifically to the needs of young transgender women living with HIV in terms of verbiage and content. The text message library was personalized in that participants could customize their 10-hour delivery timeframe, that is, intervention time period, and could personalize their delivery platform to their mobile phone or an email inbox. Several iterations occurred; the first level of modifications was made with 4 young adult transgender service providers who were given the original text-message library to adapt for young adult transgender women living with HIV. Following several revisions, the library was brought before the transspecific Community Advisory Board for the second level of modifications. The Community Advisory Board is multicultural and comprised both transgender women living with HIV and HIV-negative transgender women, program consumers, gatekeepers and stakeholders, service providers from local community-based organizations that provide services to transgender women, and members of local community planning and advocacy groups (eg, LAC Commission on HIV, Transgender Service Providers Network, and Transgender Task Force). The Community Advisory Board members reviewed and discussed every message in detail. Following the adaptation into a culturally responsive text-message library for young adult transgender women living with HIV, the research team reviewed each message and, if necessary, made minor modifications to ensure that each message had a theoretical foundation.

Text messages were based on, and equally distributed across, 3 behavioral theories: social support theory [39,40], social cognitive theory [41,42], and health belief model [43]. Social support theory encompasses instrumental, emotional, and informational supports, which have been shown to reduce stress and improve health outcomes [40,44,45]. Social cognitive theory serves to increase individuals’ self-efficacy and help develop self-regulation skills [42,46]. As low self-esteem has been associated with increased HIV risk among gender/sexual minority populations [47], increased self-esteem can serve to optimize HIV care outcomes. Health belief model states that threats to health can increase one’s likelihood of engaging in protective health behaviors [43]. Table 1 illustrates how the text message library was developed to have both a theoretical foundation and be transgenderculturally responsive.

Text messages were transmitted through gradual automation administration every day including weekends, in real time, within a 10-hour period. The automated text message delivery system was developed specifically for this study by Qualtrics, a digital survey development and administration company. Thus, participants received a text message every 5 hours (eg, at 12:00 pm, at 5:00 pm, and at 10:00 am). Each text message was unique, so participants did not receive the same scripted text message twice. The intervention was designed to be cost-efficient, sustainable, and easily scaled by community agencies throughout the United States.

### Ethical Approval

All procedures performed in studies involving human participants were in accordance with the ethical standards of the institutional and/or national research committee and with the 1964 Helsinki declaration and its later amendments or comparable ethical standards.

### Informed Consent

Informed consent was obtained from all individual participants included in the study.

**Table 1:** Sample *Text Me, Girl!* theory-based and transspecific text message library.

HIV care continuum	General message	Transspecific message	Theoretical foundation
HIV positivity/physical and emotional health	Take care of yourself.	Trans pride is taking care of yourself.	Social support theory
Linkage/retention in HIV care	See your doctor.	Protect your trans beautiful body, see your doctor.	Health belief model
Antiretroviral therapy medication adherence	Take your meds.	Take your meds, girl! You can do it!	Social cognitive theory

### Sample

The study inclusion criteria were as follows: gender identity as a woman, assigned a biological sex of male at birth, aged between 18 and 34 years, confirmed HIV-positive serostatus, ability to receive daily text messages on either a personal mobile phone or via an email account, tested HIV positive for the first time within the last 12 months or had not had an HIV care visit in the previous 6 months or had a viral load of ≥200 copies/mL on her last lab test result or not currently prescribed ART medication or was currently prescribed ART medication but did not rate her ability to take all her medications as excellent, willing and able to provide informed consent, and willing and able to comply with study requirements. Individuals were excluded if they did not meet all the eligibility criteria.

### Study Procedures

#### Recruitment

A total of 6 recruitment strategies were utilized to ensure enrollment targets were met and a diversity of participants were enrolled to foster a diverse range of sociodemographic characteristics: (1) Web-based banner ads and digital flyers placed through geo-mapping (ie, the ads only displayed in areas where study recruitment was possible) on websites and social media sites that target transgender women, (2) ads placed in print media and via an email blast for transgender women or that transgender women read; (3) street- and venue-based outreach by 2 research assistants (RAs) utilizing a modified semistructured time-space sampling methodology (ie, no sampling frame was created for random selection and sites and times were rotated on a schedule based on known migration tendencies in the community) at locations where young transgender women congregate, such as boutiques, parks, street corners, bars, clubs, hotels, nail and hair shops, and cruising boulevards, (4) study posters placed at collaborating community-based organizations containing details about how to contact an RA for further information regarding the study, (5) in-services at collaborating community-based organizations and other programs on site, and (6) participant referrals. The RAs were trained on appropriate outreach strategies and confidential screening.

#### Enrollment

At the enrollment session, potential participants were screened for eligibility, completed the informed consent process, and were administered a baseline assessment that took approximately 90 min. RAs administered screeners using computer tablets and assessments via audio computer-assisted self-interview (ACASI) to maximize accurate disclosure of high-risk and sensitive behaviors. Questionnaire Development System software was used to implement the ACASI. At the completion of the visit, an RA oriented the participant on how to maintain confidentiality and privacy on mobile devices with respect to the intervention. Participants were shown how to lock their phone, establish and use a pin code to password protect their phone or email account, and were instructed to periodically delete the intervention text messages.

#### Random Assignment

After completing the baseline assessment, participants were assigned to a condition through an urn randomization procedure. The urn randomization procedure provided balance across age (18-24 and 25-34 years), race/ethnicity (Latino/Hispanic and all other race/ethnicities), and HIV care continuum status (linked, nonlinked to HIV care). Participants were randomized into 1 of the 2 conditions: Group A—immediate text message intervention delivery (n=61) or Group B—delayed text message intervention delivery (n=69) whereby participants were delivered the text-messaging intervention after a 90-day delay period. Both groups received the same 90-day text-messaging intervention. The randomized 2-group repeated measures design assessed participants at 3, 6, 12, and 18 months post randomization.

#### Postintervention Opt-In/Opt-Out Retention and Engagement Text Messages

Immediately following the 90-day text-messaging intervention, participants were offered an opportunity to opt in to receive additional weekly text messages at a reduced schedule through their final distal follow-up evaluation. Participants in both study conditions could opt to receive the postintervention messages. Postintervention retention/engagement messages comprised 2 topic areas: (1) linkage/retention support (ie, participant received a message about linking/remaining in HIV care) and (2) ART adherence reminders (ie, participant received a message about the importance of medication adherence); a participant could opt in to any one or both of the topic areas. Each of the 2 topic areas were transmitted once a week for a maximum of 2 weekly messages. Postintervention retention/engagement messages were derived directly from the text messages already available through the Health Resources and Services Administration (HRSA)–funded UCARE4LIFE library. At each follow-up visit, participants were asked if they wanted to opt in/out to receive these additional retention/engagement text messages and were informed that they could stop the additional messages at any time by texting back *stop* to the system.

#### Incentive Schedule

Incentives comprised the following: (1) incentives for admission procedures (US $50 gift card), (2) incentives for completing follow-up assessments (US $50 gift card for completing the 3-month follow-up assessment, a bonus of US $20 for completing the 3-month follow-up assessment within an average of 5 days of the exact 3-month date, a US $50 gift card for completing the 6- and 12-month follow-up assessments, and a US $100 gift card for completing the 18-month follow-up assessment), and (3) a small gift (eg, make-up, earrings; valued at approximately US $2) when an active participant brought a potential participant to the site, and a US $20 gift card if the potential participant was eligible and enrolled (maximum of 3 eligible and enrolled participants per active participant). The total amount a participant could earn for enrolling and participating in the study was US $380.

### Evaluation Procedures

The evaluation plan for *Text Me, Girl!* was 2-fold, with both a cross-site and a local evaluation. Under the sponsorship of the HRSA Special Projects of National Significance (SPNS) Social Media Initiative**,** the Evaluation and Technical Assistance Center (ETAC) at University of California, Los Angeles, was responsible for providing technical assistance and capacity building to the local demonstration sites on clinical and program activities and leading the cross-site evaluation. The cross-site evaluation included both a behavioral and clinical assessment. The clinical assessments were carried out by a medical care provider at one of the collaborating HIV medical care clinics. Once data were collected and abstracted by the HIV medical care provider, FRI submitted the data to ETAC via a Web-based system called the ETAC Social Media Project Portal.

For the local evaluation component, the outcomes were selected based on HRSA priorities and the Los Angeles Enhanced Comprehensive HIV Prevention Plan [17]. *Text Me, Girl!* collected and reported on the required performance measures, including program-specific and required ETAC data at baseline and at follow-up data visits administered at 3 months post enrollment, and approximately 6, 12, and 18 months post enrollment. The project director, research coordinator/evaluator, and RAs were trained in administration of the assessments and on the procedures to be used for data submission to HRSA.

In addition to the local and cross-site assessments, process evaluation and study monitoring were also carried out as part of the overall evaluation of *Text Me, Girl!*. Process evaluation procedures included the following: (1) formative evaluation of the study to update and refine study components that were responsive to the needs of young HIV-positive transgender women, (2) process monitoring of the delivery of the study, and, (3) process evaluation of successful linkage, engagement, and retention among participants. An intervention exposure (IE) question was administered as part of the reporting protocol. Using the study mobile phone, an RA sent a monthly IE text message to each participant on a Wednesday afternoon between 3:00 pm and 7:00 pm. The IE text message was as follows: “In the past month, how many of the text messages did you read? Please text back one of the following: 1) none, 2) some but less than half, 3) about half, 4) a lot but not all, or 5) all.”

Outcome data were generated through 2 mechanisms: (1) assessments performed onsite at baseline and at 3, 6, 12, and 18 months post baseline and (2) electronic medical record (EMR)/electronic health record (EHR) data abstraction. In each case, data were imported from delimited text format (ie, .csv, the native format for the cloud-based data storage, scanned paper assessments, and extracted EMR/EHR data) into Stata v13SE (StataCorp).

### Measures

All primary outcomes came directly from the US Health and Human Services (HHS) Common HIV Indicators for the HRSA/SPNS project (FOA HRSA-15-029). Utilization of these collection procedures provided data that could be compared with other clinical services and programs serving high-risk populations and populations that are underrepresented in HIV care. As the ETAC cross-site evaluation procedures and measures are described in detail elsewhere [48], only the local evaluation measures are described below.

#### Local Evaluation for Text Me, Girl! Only

The Local Evaluation was a behavioral assessment that included questions in the following domains: gender identity and presentation including hormone use, sexually transmitted infections, sexual behaviors, and HIV self-efficacy. The assessment measures that were used as part of the local (not cross-site) evaluation component of *Text Me, Girl!* are described below.

##### HIV Health Assessment

Originally developed by the principal investigator (PI) CJR, for an intensive prevention case management intervention, this instrument records demographics (eg, sexual identity, age, and racial/ethnic identities), educational attainment, housing status, access to insurance, HIV treatment status (including position in the HIV care continuum), HIV medication status (including medication type and dose) and self-reported ART adherence, and barriers to receiving HIV care [49]. Only the domains specific to the outcome measurements of this study that did not duplicate the ETAC cross-site evaluation were used.

##### The Los Angeles Transgender Health Survey

This instrument was developed by CJR and colleagues in 1997 in consultation with members of the LAC transgender women communities and appropriately updated in the interim years. The instrument comprises 7 modules: screening, sociodemographic characteristics, health care access and medical history, sexual behaviors (at all stages of gender transition and gender confirmation surgery), drug and alcohol use, legal and psychosocial issues, and HIV prevention [50]. Only the domains specific to the outcome measurements of this study and those that did not duplicate the HIV health assessment or the ETAC cross-site evaluation were used.

##### HIV Treatment Adherence Self-Efficacy Scale

The HIV treatment adherence self-efficacy scale (HIV-ASES) comprises 12 items assessing participants’ self-efficacy to adhere to their ART medication regimen (0=cannot do it at all to 10=completely certain can do). Cronbach alpha for the measure is robust (routinely >.90). The HIV-ASES is lightly adapted to the target populations’ lower average literacy level and refers specifically to participants’ level of confidence that they can maintain adherence. The scale assessed how confident participants were to integrate treatment into daily routines, stick to a treatment plan even if it was disruptive or they felt unwell, continue treatment plan even if the level of T cells drops significantly, and get something positive out of participation in treatment even if the medication did not seem to improve their health [51].

### Statistical Analyses

Table 2 provides all evaluation outcomes and their chosen measurement models. Power calculations are based on assumptions of alpha=.05 (2-tailed tests), a sample size of 130 HIV-positive young transgender women, and a power standard of 0.80. It was calculated that logistic regression analyses of primary outcomes would be able to find a minimum detectable odds ratio of 2.98 for linkage/uptake of ART and achievement of an undetectable viral load (the primary outcomes of interest).

**Table 2:** Study outcomes and measurement models.

Primary outcome	Measurement model
Linkage to HIV primary care (self-report and EMR^a^/EHR^b^ abstraction)	0= Participant did not attend HIV medical visit within 3 months of enrollment; 1= Participant attended within the first 3 months
Prescribed ART^c^ medication (self-report and EMR/EHR abstraction)	0=Participant is not prescribed HIV medication; 1=Participant is prescribed HIV medication
Retention in HIV primary care (self-report and EMR/EHR abstraction)	0=Participant did not attend a second HIV care appointment after the initial 3 months; 1=Participant attended an additional HIV primary care visit after the initial 3 months
ART adherence (self-report)	Ordinal scale (Likert)
Viral load monitoring (self-report and EMR/EHR abstraction)	Binary (0=>200 copies/mL, 1=<200 copies/mL)

^a^EMR: electronic medical report.

^b^EHR: electronic health report.

^c^ART: antiretroviral therapy.

All regression analyses assess assumptions of normality and model fit. In addition to the primary outcomes listed in Table 2, participants were assessed for changes in their HIV-related self-efficacy from baseline to follow-up evaluations. The HIV-ASES scale was used to assess HIV self-efficacy, producing continuous values suitable for ordinary least squares (OLS) regression. In addition, if during implementation, a sufficient number of participants opted in to receive one or more of the postintervention retention/engagement text messages available through final distal follow-up evaluation, additional analyses were carried out to estimate the observed effect(s) of receiving these postintervention text messages.

All calculations of detectable effects for primary outcomes were based on expected cultural/demographic subgroup analyses premised on known social determinants of risk (eg, racial/ethnic identity, language and health insurance) and are displayed in Table 2. Secondary analyses related to HIV self-efficacy were analyzed using OLS regression and should produce detectable effects at *f*^2^=0.067. Given these values, detectable effects of all analyses should, if benchmarked, fall in the *small to moderate* to *moderate* ranges. Given an expected follow-up rate of at least 85% (111/130) at each time point, associations between primary outcomes and optional postintervention retention/engagement messages should produce minimum detectable effects exhibiting a 3% (repeated measures survival analysis testing time-based dichotomous outcomes, eg, linkage) to 18% (OLS regression for secondary analyses, eg, HIV-related self-efficacy scores) increase in detectable effect size (ie, outcomes will be more difficult to detect), resultant from participant attrition.

Primary outcome analyses comprised bivariate contrasts of outcomes over time (ie, HIV care continuum outcomes at baseline and each follow-up, with differences tested via *t* test and chi-square analyses) and multivariate analyses of these same outcomes regressed on random study arm assignment, self-reported exposure to theory-based text messages, and time. Observations were nested within participants, and sociodemographic controls were tested for inclusion during sensitivity testing of the models. Secondary outcomes included sexual risk behaviors, substance use, self-efficacy, and changes in gender identity over time.

## Results

Recruitment began on November 18, 2016, and the first participant was enrolled on December 16, 2016; enrollment closed on May 31, 2018. Intervention delivery ended on November 30, 2018, and follow-up evaluations will conclude on August 31, 2019. Primary outcome analyses will begin immediately following the conclusion of the follow-up evaluations.

## Discussion

*Text Me, Girl!* was designed to meet the needs of underserved young transgender women living with HIV/AIDS. Young transgender women living with HIV experience a range of barriers that challenge their ability to be successfully linked and retained in HIV care. Text messaging is a communication platform well suited for engaging young transgender women in HIV care because it is easily accessible, widely used, private, portable, and inexpensive. *Text Me, Girl!* aimed to improve HIV care continuum outcomes among young transgender women by providing culturally responsive text messages to promote linkage, retention, and adherence, with the ultimate goal of achieving viral suppression. The *Text Me, Girl!* text message library is readily scalable and, if successful, can be adapted for application with other hard-to-reach populations.

## Abbreviations

ACASI: audio computer-assisted self-interview
ART: antiretroviral therapy
EHR: electronic health record
EMR: electronic medical record
ETAC: Evaluation and Technical Assistance Center
FRI: Friends Research Institute
HHS: Health and Human Services
HIV-ASES: HIV Treatment Adherence Self-Efficacy Scale
HRSA: Health Resources and Services Administration
IE: intervention exposure
LAC: Los Angeles county
OLS: ordinary least squares
PI: principal investigator
PLWH: people living with HIV
RA: research assistant
SPNS: Special Projects of National Significance

## Appendix

Multimedia Appendix 1 Peer-reviewer report from the Health Resources and Services Administration.
