# Supplementary material for: Text Messaging to Improve Linkage, Retention, and Health Outcomes Among HIV-Positive Young Transgender Women: Protocol for a Randomized Controlled Trial (Text Me, Girl!)
Source: JMIR Res Protoc. 2019 Jul 29;8(7):e12837. doi: 10.2196/12837 (PMC6690158; doi:10.2196/12837)
Supplement: Multimedia Appendix 1 [file researchprotocols_v8i7e12837_fig.pdf]

Health Resources and Services Administration
HRSA-15-029

HAB: Use of Social Media along the HIV Care Continuum - Demonstration Sites
Objective Review Committee Final Summary Statement

Score: 100

Application Number: 128781
Application Name: FRIENDS RESEARCH INSTITUTE, INC
State: MD City: Baltimore

Criterion 1: NEED

Strength:

The applicant organization provides a thorough introduction to the proposed social media intervention to improve linkage and retention in HIV care.

The applicant organization demonstrates extensive knowledge of the literature regarding problems along the HIV Care Continuum specific to transgender women and social media concepts.

The application clearly explains the specific needs of the target population, HIV-positive young transgender women, linking data to a model of HIV care continuum and the potential impact social media has on this group.

The applicant organization clearly demonstrates how it will use behavioral theory-based, population-specific text messaging to address the specific needs and behaviors of the target population.

Weakness:

None

Criterion 2: RESPONSE

Strength:

The applicant organization depicts its structured approach to implement the proposed intervention and document and evaluate its finding.

The proposed intervention is theory-based, and the application demonstrates logical evidence that each of the three theories of Social Support, Social Cognition, and Health Beliefs are appropriate for developing text messages.

The application comprehensively details staff training as well as multimodal communication and recruitment which will enhance both enrollment and retention.

Focus groups and involvement of the community advisory board in text message development will strengthen the acceptability of messaging.

The applicant organization clearly explains how providing patients with choices in social media platforms will increase participation with the target population.

The application clearly describes a system of five outreach strategies, both conventional and non-traditional, and includes participation in numerous collaborating community-based organizations.

Weakness:

The application does not adequately explain whether participants are able to interact with program staff through text messaging or other platforms.

Based on the application's proposed timeline, participants will continue to be recruited until the end of Year 3. It is unclear whether those participants will be able to complete the proposed 24-month post follow-up surveys in the grant time period.

Criterion 3: EVALUATIVE MEASURES

Strength:

The applicant organization demonstrates its ability and capacity to participate in the multi-site evaluation and its strategy to evaluate the proposed intervention and service delivery.

The application clearly delineates what outcomes will be measured in Table 4 and specific goals for improvement.

The applicant organization clearly indicates that it has established routines, protocols, and staff to coordinate with the Evaluation and Technical Assistance Center (ETAC) for multi-site evaluation activities.

Weakness:

None

Criterion 4: IMPACT

Strength:

The applicant organization clearly addresses the impact/outcomes and how the data will be used to inform program development and service delivery.

The applicant organization has clear experience working collaboratively with the Evaluation and Technical Assistance Center (ETAC) and specifies plans for disseminating results in multiple venues at multiple levels.

The applicant organization will employ methods to achieve high follow-up rates used in similar projects that produced impressive participation.

Weakness:

The application does not adequately detail how the intervention will be sustained beyond the funding period, given that the text messaging program is run by a subcontractor, and there is a financial cost to continuing the program.

Criterion 5: RESOURCES/CAPABILITIES

Strength:

The two transgender female research assistants in particular are integral to the success of recruitment and enrollment by employing members of the community of interest.

The applicant organization successfully demonstrates that it has current staffing capacity and infrastructure to conduct these project as well as closely related, successful projects related to social media and young transgender women who are HIV positive.

Weakness:

None

Criterion 6: SUPPORT REQUESTED

Strength:

The application includes a detailed breakdown of costs and indicates appropriate use of resources. In particular, the changes in personnel costs based on who will be needed at what time, and increases based on cost of living increase, are well thought out.

The staffing plan clearly demonstrates strong experience and an appropriate level of effort to perform the activities outlined in the work plan.

Weakness:

The application does not adequately specify why the requested items and costs listed for Risk Reduction Supplies are different between Year 1 ($250) and Years 2-4 ($1500).
